# The effect of a new lifetime-cardiovascular-risk display on patients’ motivation to participate in shared decision-making

**DOI:** 10.1186/s12875-018-0766-x

**Published:** 2018-06-09

**Authors:** Nikita Roman A. Jegan, Sarah Anna Kürwitz, Lena Kathrin Kramer, Monika Heinzel-Gutenbrunner, Charles Christian Adarkwah, Uwe Popert, Norbert Donner-Banzhoff

**Affiliations:** 1 Department of General Practice and Family Medicine, Philipps-University Marburg, Karl-von-Frisch-Str. 4, 35032 Marburg, Germany; 2 MH Statistical Consulting, Marburg, Germany; 3 Department of Health Services Research, Maastricht University, Maastricht, the Netherlands; 4 Department of General Practice, Georg-August-University, Göttingen, Germany

**Keywords:** Shared decision-making, Risk, Risk assessment, Decision aid, Comprehension

## Abstract

**Background:**

This study investigated the effects of three different risk displays used in a cardiovascular risk calculator on patients’ motivation for shared decision-making (SDM). We compared a newly developed time-to-event (TTE) display with two established absolute risk displays (i.e. emoticons and bar charts). The accessibility, that is, how understandable, helpful, and trustworthy patients found each display, was also investigated.

**Methods:**

We analysed a sample of 353 patients recruited in general practices. After giving consent, patients were introduced to one of three fictional vignettes with low, medium or high cardiovascular risk. All three risk displays were shown in a randomized order. Patients were asked to rate each display with regard to motivation for SDM and accessibility. Two-factorial repeated measures analyses of variance were conducted to compare the displays and investigate possible interactions with age.

**Results:**

Regarding motivation for SDM, the TTE elicited the highest motivation, followed by the emoticons and bar chart (*p* < .001). The displays had no differential influence on the age groups (*p* = .445). While the TTE was generally rated more accessible than the emoticons and bar chart (*p* < .001), the emoticons were only superior to the bar chart in the younger subsample. However, this was only to a small effect (interaction between display and age, *p* < .01, *η*^*2*^ = 0.018).

**Conclusions:**

Using fictional case vignettes, the novel TTE display was superior regarding motivation for SDM and accessibility when compared to established displays using emoticons and a bar chart. If future research can replicate these results in real-life consultations, the TTE display will be a valuable addition to current risk calculators and decision aids by improving patients’ participation.

**Electronic supplementary material:**

The online version of this article (10.1186/s12875-018-0766-x) contains supplementary material, which is available to authorized users.

## Background

Risk calculators for cardiovascular events (CE), such as myocardial infarction or stroke have been recommended by German [1], American [2] and British [3] guidelines to aid patients in understanding their quantitative risk and the possible benefits of various interventions. They make an individual calculation of risk and the recommendation of treatments, such as lipid-lowering medication possible. When used as part of a decision aid, such as the arriba™ protocol, risk calculators can improve patients’ knowledge, increase participation in the decision-making process (shared decision-making, SDM [4, 5]) and lead to decisions that are more congruent to their values [6].

Multivariate risk functions provide the absolute 5- or 10-year risk for suffering a CE. Apart from the numerical result, cardiovascular risk calculators may also present the risk in different graphical formats [3]. Icon arrays depict risk as a natural frequency (X of 100). In this type of display, emoticons are often used to communicate the number of people with the outcome of interest. Other frequently used forms are vertical bar charts comparing the individual risk to the mean or median risk, or distribution graphs of the risk across all age groups. These formats vary in complexity. Therefore, they should be chosen according to the level of graphical literacy and preference of each patient [3, 7].

Decision-making based on the absolute risk for a defined timespan, such as ten years, has been criticized [8–10]. Younger individuals with high-risk behaviour, such as smoking or unhealthy diet, have relatively small absolute risks for CEs, although their lifetime risk is high [8–12]. This stems from the fact that absolute risk calculations are largely influenced by age [10]. When absolute risks are calculated, the benefits of early interventions most relevant to this age group such as diet and regular physical activity do not become evident. Therefore, risk calculators based only on absolute risk do not provide adequate information for this age group to help them make well-informed decisions. To address this problem, research recommends using lifetime-risk calculations [3, 8, 9] or, more specifically, the number of years free from a CE and the number of years gained by an intervention, i.e., time-to-event (TTE) [10].

A well-established and evaluated risk calculator and decision aid in Germany is arriba™ [13–17]. It uses a modified Framingham formula to calculate the absolute 10 year risk for a CE [18, 19]. So far, arriba™ has provided three graphical displays with increasing complexity to inform patients about their risk: 1) a 10 by 10 field of icon arrays portrayed as emoticons; 2) a vertical bar chart showing the individual risk and the median risk of the same age and gender group; 3) a distribution graph of the risk across all age groups. Arriba™ is predicated on the philosophy of SDM with an explicit script to be used during consultation. Following this protocol results in higher satisfaction, higher participation and lower decisional regret with primary care patients [14].

We have developed a TTE display to be incorporated into arriba™. It shows in how many years and at what age a CE is likely to occur in an individual patient. A horizontal bar depicts the total lifespan. The point in time with the highest risk for an event is marked by a different colour. The possible gain by interventions is shown in a second horizontal bar. In order to present individualized TTE predictions, we developed a Markov-based microsimulation model based on cardiovascular risk factors.

This new display has already been compared qualitatively with the established arriba™ displays in a preliminary study (Kürwitz et al.: Playing on fears - Family physician's comparative evaluation of a new risk format presenting cardiovascular risk, in preparation; Kürwitz et al.: Such a display can be hard enough!- Patients evaluation of a new risk format: cardiovascular risk presented as time-to-event, submitted). In this study, different displays based on fictional case vignettes were shown to patients and general practitioners (GPs). Respondents were asked to comment on comprehensibility, risk perception, motivation to participate in SDM, and ethical defensibility. The new TTE display received the most favourable feedback, with emoticons and a bar chart following and the distribution graphs lagging behind.

In this article, we first present the quantitative evaluation of these three displays regarding their ability to elicit motivation for SDM in patients. Second, we test the hypothesis that the TTE display leads to a higher motivation for SDM than the other two displays in younger patients when compared to older patients. This hypothesis is based on the assumption, that an increased lifetime risk of young patients should become more apparent in the TTE display, creating a higher subjective feeling of risk. In other words, we evaluate a possible interaction with the age of responding patients. Finally, we asked patients which of the three displays was the most understandable, helpful and trustworthy (‘accessible’).

## Methods

### Design

**Fig. 1:**
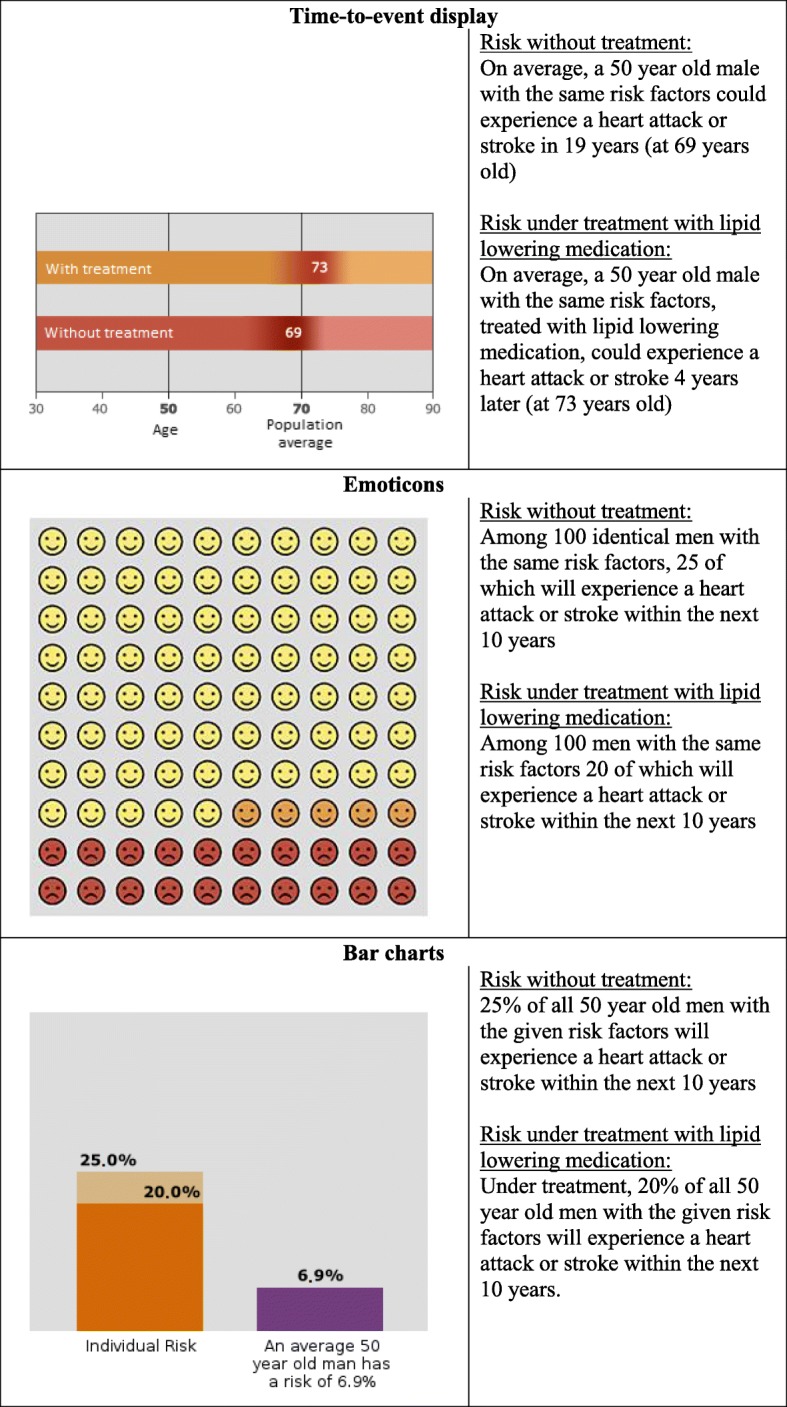
Risk displays used in the study

We contacted 100 GPs in the northern region of Hesse, Germany for participation. A total of 30 agreed to participate while 19 declined. The remaining 51 did not reply. Those who agreed were asked to consecutively recruit 20 patients among those presenting for a health check. In case the recruitment goal could not be reached by a single GP, patients visiting as part of the diabetes type 2 Disease Management Program were also allowed to be included. Recruited patients had to be between 35 and 70 years old, with 35 being the earliest eligible age for an adult health check provided by public healthcare in Germany. After giving their written consent, patients completed a questionnaire, which covered sociodemographic characteristics and their reactions to different risk presentation formats. They were introduced to one of three fictional case scenarios, namely low, medium or high cardiovascular risk). The low risk vignette portrayed a 47-year-old healthy, non-smoking female with normal weight, consulting her GP after reading a news article about cholesterol. The medium risk vignette portrayed a 50 year old smoking male (12 cigarettes per day), physically inactive, and with a high fat and high sugar diet being sent to his GP by his worried wife. Finally, the high-risk vignette described a 50-year-old heavy-smoking male with a high workload and imbalanced diet, reporting to his GP after hospitalisation due to a heart attack. The vignette was then followed by the three displays (emoticons, bar chart and TTE, Fig. 1) showing the cardiovascular risk and the achievable change by lipid lowering (statin) treatment. Each display was presented along with a verbal description of the displayed risk. The patients were instructed to answer the questions as if they were the person described in the fictional case.

The sequence of displays was varied randomly to avoid systematic sequence effects. Moreover, low, medium, and high-risk scenarios were evenly distributed in each practice. Finally, the order of fictional cases and permutations of displays was varied systematically from practice to practice.

After six weeks, we contacted a random subsample again to evaluate the test-retest reliability of the scale measuring motivation for SDM.

### Measures

#### Sample description

We assessed sociodemographic patient data by asking about age, gender, education, and migrant status [20]. GPs were asked to specify known cardiovascular risk factors for each patient and state if the patient has been exposed to counselling with arriba™ before.

#### Motivation for SDM

Due to the lack of scales measuring the motivation to participate in SDM [21, 22], we created eight items asking about the motivation to perform behaviours that are crucial for the participation in making a well-informed decision. Patients were asked to rate their agreement with each statement on a five-point Likert scale. Then, we calculated the means across all items for each display. Internal consistency and retest-reliability-coefficients (six week interval) were initially moderate (Cronbach’s α = .589–.684, r_tt_ = .485–.834). Eliminating two items improved the results (Cronbach’s α: .867–.877, r_tt_ = .562–.890) and led to the six- item solution we used for further analysis (Table 1).

**Table 1:** Motivation for shared decision-making scale; final six-item version (translated from German for the purpose of this article)

If I was the patient being shown the information…	Not at all (1)	Not Likely (2)	Partially (3)	Likely(4)	Very much (4)
1. …I would be motivated to think further about my risk.					
2. …I would be motivated to request further information from my family doctor.					
3. …I would be motivated to use other sources to learn more about my risk.					
4. …I would feel sufficiently informed by the display to make a decision for or against an intervention.					
5. …I would be motivated to talk with my doctor about the decision for or against therapy.					
6. …I would be sufficiently informed to decide together with my doctor whether I should receive treatment.					

#### Accessibility

To measure the subjective accessibility of the information, we used the accessibility scale developed by Gaissmaier and colleagues. The scale includes five items asking for subjective judgements regarding comprehensibility, usefulness, seriousness, and intuitive accessibility on a five-point Likert scale [23].

#### Sample size calculation and statistical analysis

We calculated the required sample size a priori with G*Power (Ver.3.1.5) [24]. Our main hypothesis was tested with a repeated measures analysis of variance with three measurements (displays) and two groups (young vs. old patients, median split) for the main outcome “motivation for SDM”. Assuming a small effect-size of Cohen’s *ƒ* = 0.10 [25] for the interaction term, we calculated a required sample size of *n* = 324 to achieve an α-error of 5% and a power (1-β) of 0.8. Taking into account a 90%participation rate and a 10% data loss due to missing values, we would need to approach 405 patients.

All calculations were conducted with IBM SPSS (Version 21) [26]. We calculated means, standard deviations and frequencies for variables of interest. After splitting the sample at the median age, we compared the two age groups regarding demographic variables and risk factors with χ^2^-, Fisher’s exact and t-tests. The internal consistency of the motivation-for-SDM scale was determined by calculating Cronbach’s α coefficients [27] and the test-retest-reliability was quantified by Pearson correlations. The comparison of the displays in terms of motivation for SDM and possible differential influences on the age groups was tested with a two-factorial repeated measures analysis of variance. We used the same procedure to analyse the secondary outcome “accessibility”. In case of significant main or interaction effects, we calculated pairwise comparisons with post-hoc Scheffé tests to account for multiple testing.

## Results

### Sample characteristics

**Fig. 2:**
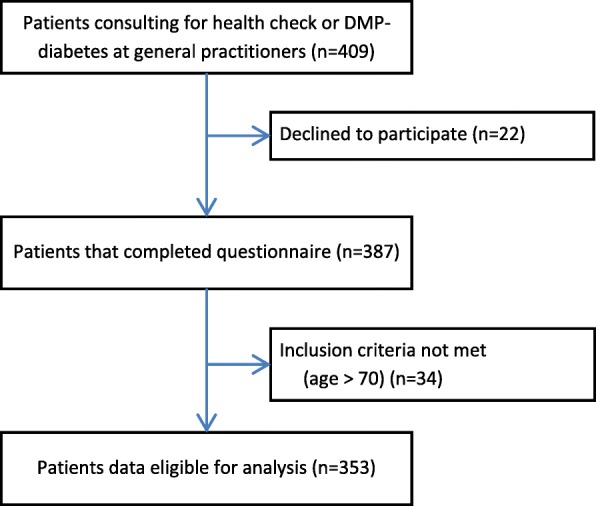
Study flowchart. DMP = disease management program

In total, 409 patients were asked for participation, of which 22 patients declined. The remaining 387 patients completed the questionnaire immediately after consultation. However, 34 of them were older than 70 years and were subsequently excluded from the analysis (Fig. 2). The baseline characteristics of the analysed sample (*n* = 353) along with the subsamples after splitting at the median age (52 years) can be seen in Table 2. Young and old patients differed in educational background (a higher proportion of basic education and lower proportion of higher education in the older patients), as well as in the number of cardiovascular risk factors (both *p*-values < .001). When calculating the statistical tests, we had to exclude a small number of cases due to missing values in the outcomes (motivation for SDM: 20 cases; accessibility: 46 cases). For each outcome, we compared the patients with complete data sets to those with incomplete data sets. No statistical differences could be found except for migrants who had more frequently missing values than Germans (motivation for SDM: 16.7% vs. 4.7% (*p* = .021); accessibility: 26.7% vs. 11.4% (*p* = .038); *n* = 346 due to seven missing values in immigration data, see Additional file 1). Furthermore, we compared patients receiving the small, medium, and high-risk case vignettes to control for possible group imbalance and found no differences in any of the demographic variables (see Additional file 2).

**Table 2:** Baseline characteristics of patient sample and subsamples after splitting at the median age (52 years)

Variable	Total sample	Young patients	Old patients	Young vs. old
	(*n* = 353)	(*n* = 178)	(*n* = 175)	*p*-value
Female, *n (%)*	197 (55.8%)	112 (62.9%)	85 (48.6%)	.007^a^
Mean age in years*, *M (SD)*	53.5 (9.0)	45.9 (4.7)	61.2 (4.8)	<.001^b^
Migrant status, *n (%)*				
No (German)	316 (89.5%)	157 (88.2%)	159 (90.9%)	.495^a^
Yes	30 (8.5%)	16 (9.0%)	14 (8.1%)	
Insufficient data	7 (2.0%)	5 (2.8%)	2 (1.1%)	
Education*, *n (%)*				
Basic education (up to 9 years)	114 (32.4%)	36 (20.3%)	78 (44.6%)	< .001^a^
Medium education (10–11 years)	138 (39.2%)	81 (45.8%)	57 (32.6%)	
Higher education (12 years and more)	100 (28.4%)	60 (33.9%)	40 (22.9%)	
Reason for Consultation, *n (%)*				
Health check	337 (95.5%)	175 (98.3%)	162 (92.6%)	.009^a^
Disease management for diabetes	16 (4.5%)	3 (1.7%)	13 (7.4%)	
Number of risk factors besides age*, *M (SD)*	0.56 (0.8)	0.28 (0.6)	0.83 (0.94)	<.001^b^
Existing risk factors				
Existing *c*oronary heart disease	12 (3.4%)	3 (1.7%)	9 (5.1%)	.085^c^
Prior myocardial infarction	8 (2.3%)	2 (1.1%)	6 (3.4%)	.172^c^
Prior stroke	14 (4.0%)	2 (1.1%)	12 (6.9%)	.006^c^
Peripheral artery disease	7 (2.0%)	1 (0.6%9	6 (3.4%)	.066^c^
Diabetes	40 (11.3%)	7 (3.9%)	33 (18.9%)	<.001^a^
Hypertension	115 (32.6%)	35 (19.7%)	80 (45.7%)	<.001^a^
Contact with arriba™ prior to examination, *n (%)*	54 (15.3%)	23 (12.9%)	31 (17.7%)	.211^a^

*SD* standard deviation, *CVD* cardiovascular disease;

* = difference between old and young patients is significant by *p* < .001;

^a^ = *χ*^*2*^-test; ^b^ = t-test; ^c^ = Fisher’s exact test

### Primary and secondary outcomes

**Fig. 3:**
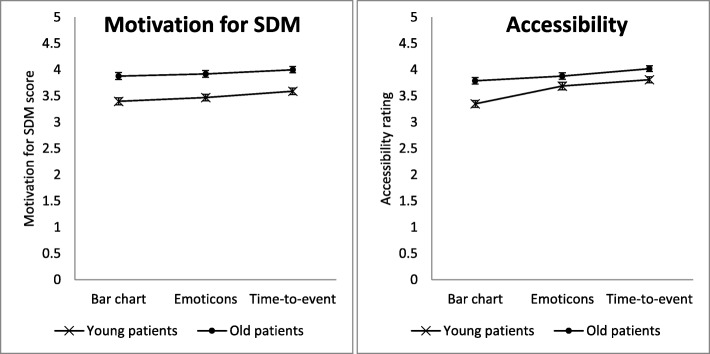
Motivation for SDM and accessibility ratings of young and old patients for each display. Patient sample was split at the median age (52 years); SDM = shared decision-making

Examining the primary outcome “Motivation for SDM”, all three display types differed from each other (medium to large effect size; all results shown in Table 3 and Fig. 3). The TTE display received significantly higher ratings than emoticons and bar charts. In addition, the emoticons were rated higher than the bar chart. This applied irrespective of age (interaction effect age * display not significant). In general, older patients felt a higher motivation for SDM than younger patients, irrespective of the display type (small to moderate effect size).

**Table 3:** Comparison of young and old patients’ ratings of the three displays

Variable / Sample	Bar chart	Emoti-cons	Time-to-event	Total	Display	Age	A*D^b^	Post-hoc tests^c^
	M (SD)	M (SD)	M (SD)	M (SD)	*p*-value (η^2 a^)	*p*- value (η^2 a^)	*p*- value (η^2 a^)	
Motivation for SDM^d^								
Young (*n* = 169)	3.40 (0.89)	3.47 (0.86)	3.59 (0.83)	3.49 (0.79)	<.001 (0.035)	< .001 (0.084)	.445 (0.002)	T > E, E > B, T > B
Old (*n* = 164)	3.88 (0.81)	3.92 (0.77)	4.00 (0.74)	3.94 (0.70)				
Total (*n* = 333)	3.64 (0.89)	3.69 (0.85)	3.79 (0.81)					
Accessibility^d^								
Young (*n* = 155)	3.35 (0.88)	3.69 (0.79)	3.81 (0.73)	3.62 (0.65)	< .001 (0.102)	< .001 (0.045)	.005 (0.018)	T > E, E > B, T > B
Old (*n* = 152)	3.79 (0.76)	3.88 (0.76)	4.02 (0.68)	3.90 (0.64)				T > E, T > B
Total (*n* = 307)	3.57 (0.85)	3.79 (0.78)	3.92 (0.71)					

*SDM* shared decision-making, *M* mean, *SD* standard deviation, *E* emoticons, *B* bar chart, *T* time-to-event display

^a^η^2^ = effect size; 0.01 = small effect; 0.06 = medium effect; 0.14 = large effect

^b^Interaction between age group and display. Significance means that the displays’ ratings relate differently to each other in each group

^c^Post-hoc Scheffé pairwise comparisons of display types in case of significant main effect or interaction: > means significantly higher rating

^d^Range 1–5; higher numbers reflect higher perceived ability to motivate patients to participate in SDM, or a higher perceived accessibility, respectively

The analysis for the secondary outcome “accessibility” showed significant results for age and display (Table 3 and Fig. 3). Again, older patients rated all displays higher than younger patients, but this time with a smaller effect. In addition, there was an interaction effect between age and display: both of the two patients groups (young and old) rated the TTE display higher than emoticons and the bar chart. The latter, however, was rated lower than the emoticons by the younger patients. The effect size for this interaction was small.

## Discussion

Our results suggest that the displays differently influence the patients’ motivation to participate in the SDM process. The novel TTE display showed the highest potential, followed by emoticons and the bar chart. The differences between displays, however, were small. Older patients generally reported a higher motivation for SDM, but there was no difference in how the displays affected motivation for SDM among older and younger patients. It is notable that the order the displays are ranked (TTE first, followed by emoticons and the bar chart) is the same for “motivation for SDM” and “accessibility” (see Fig. 3).

To date, literature on the effect of TTE predictions on patient decision-making is scarce. In fact, while looking for explanations for the different effects of the displays on the motivation to participate in SDM, we were unable to find any previous work exploring this issue. However, existing literature shows that TTE formats are superior to other formats in terms of a subjective perception of understandability of the presented risk information [28–30].

In a Danish study, participants were asked how difficult it was for them to understand a presented information about a fictional drug treatment postponing heart attacks. The information was presented in a verbal TTE format. There was no comparison with another risk format, but overall level of understandability was high with 81% of all participants judging the information as “not difficult to understand” [28].

Another study compared the effects of a hypothetical osteoporosis intervention either presented in numbers-needed-to-treat, or a verbal TTE format, presenting the duration a hip fracture could be postponed. The TTE information was associated with lower subjective uncertainty about the meaning of the presented information [29].

A web-based study presented the benefit of a fictional antibiotic in one of several formats to healthy individuals [30]. Among them was a TTE display that showed the duration of symptoms with and without treatment. It was judged the easiest format to understand.

In summary, these results show that patients prefer risk information presented in a TTE format. Moreover, it was perceived as easier to understand than traditional absolute risk information. It can be assumed that an improved feeling of understanding translates into higher enablement to form a decision and, therefore, a higher motivation to participate in the decision-making process. This might be the case for our results as well.

As demonstrated in Fig. 2, the pattern showing the displays ranking is identical to that for “motivation for SDM” and “accessibility”. In this way, the TTE display might elicit the highest motivation to participate in SDM because the feeling of understanding is also highest in the display that is judged as easier to understand. This hypothesis, however, cannot be proven with our results. A future study could include a measurement of enablement or certainty and examine how this is related to the displays and the motivation to participate in SDM.

The fact that older patients rated the displays more accessible and felt more motivated by them in our study can be explained by differences in experience dealing with risk. Since the older patients had significantly more risk factors and, therefore, a longer history of consultations, they might have more background knowledge and thus be more involved and motivated to participate in the decision regarding their health [31].

The lack of interaction between the displays and age might have methodical reasons. Patients were either presented a low, medium, or high-risk scenario, all depicting cases of the same age group (49–50 years old). They were instructed to imagine themselves in the place of the fictional person and answer the questions as if they were that person. Therefore, even if the groups differed in the actual age of the participants, they did not differ in the age of the case vignettes with which participating patients were expected to identify with. As a result, there might have actually been no difference in subjective risk between the groups and, therefore, no interaction effect. In order to avoid this effect, future investigations based on vignettes should use vignettes featuring different age groups and be compared irrespective of the actual age of the participants. On the positive side, this indicates that patients were able to understand and follow the instructions as we intended.

This study has several limitations. First, even though patients were able to identify with the fictional cases, their involvement might be different when confronted with their own risk and related decisions. Second, for technical reasons we presented the risk information in a paper-based form with a static picture for each display. In reality, the information is presented on a computer screen and can be changed interactively to show differences in risk due to interventions. This could actually lead to a higher participation since the patients can engage more in the process. Third, since there is a lack of scales measuring motivation to participate in SDM, we developed a new scale for this study. Although we achieved good reliability, the actual validity of the scale remains to be shown since we did not use other scales to measure divergent and convergent validity. Ideally the scale would be validated in real-life consultations together with ratings of the actual behaviour and involvement in the decision-making [32]. In our point of view, despite these limitations, the following conclusions can be drawn from our findings.

## Conclusions

In a survey of primary care patients, a TTE display increased motivation to participate in decisions regarding cardiovascular risk modification. In this, TTE displays were superior to absolute risk displays such as emoticons and bar charts. Since we asked patients to assess fictional cases, future studies should compare risk displays with patients being informed about their own risk and having to make real life decisions. Since patients also judged the TTE display as easier to understand, more useful and more serious (i.e. more accessible) than emoticons and bar charts, the new TTE display is be a valuable addition to current risk calculators and decision aids.

## Additional files

Additional file 1: **Table S1** and **S2**. Comparison of patients with complete and incomplete datasets in the motivation for shared decision-making scale and the accessibility scale. (DOCX 14 kb)

Additional file 2: **Table S3**. Comparison of patients randomly receiving either the low, medium or high cardiovascular risk case vignette. (DOCX 13 kb)

## Abbreviations

CE: General Practitioners
SDM: Shared Decision-Making
TTE: Time-to-event
