# Supplementary material for: The effect of a new lifetime-cardiovascular-risk display on patients’ motivation to participate in shared decision-making
Source: BMC Fam Pract. 2018 Jun 9;19:84. doi: 10.1186/s12875-018-0766-x (PMC5994251; doi:10.1186/s12875-018-0766-x)
Supplement: Supplementary file 1 — Table S1 and S2. Comparison of patients with complete and incomplete datasets in the motivation for shared decision-making scale and the accessibility scale. (DOCX 14 kb) [file 12875_2018_766_MOESM1_ESM.docx]

Supplemental table 1 Comparison of groups with complete and incomplete datasets in the motivation for SDM scale

| Variable | Motivation for SDM scale complete  (n = 333) | Motivation for SDM scale incomplete  (n = 20) | p-value |
| --- | --- | --- | --- |
| Gender, *n* (%)  Female  Male | 188 (95.4%)  145 (92.9%) | 9 (4.6%)  11 (7.1%) | .316^a^ |
| Mean age in years*, *M (SD)* | 53.5 (9.0) | 54.0 (10.0) | .793^b^ |
| Migration, *n (%)*  No (German)  Yes  Insufficient information | 301 (95.3%)  25 (83.3%)  326 (94.2%) | 15 (4.7%)  5 (16.7%)  20 (5.8%) | .021^c^ |
| Education*, *n (%)*  Basic Education (up to 9 years)  Medium Education (10-11 years)  Higher Education (12 years and over) | 109 (95.6%)  129 (93.5%)  94 (94.0%) | 5 (4.4%)  9 (6.5%)  6 (6.0%) | .757^a^ |
| Reason for Consultation, *n (%)*  Health Check  Disease Management for Diabetes | 319 (94.7%)  14 (87.5%) | 18 (5.3%)  2 (12.5%) | .227^c^ |
| Number of risk factors besides age*, *M (SD)* | 0.6 (0.8) | 0.5 (0.8) | .559^b^ |
| Contact with arriba prior to examination, *n (%)*  Yes  No | 279 (93.3%)  54 (100%) | 20 (6.7%)  0 (0%) | .053^c^ |

SDM = shared decision making

^a^= *χ^2^*-test; ^b^ = t-test; ^c^ = Fisher’s exact test

Supplemental table 2 Comparison of groups with complete and incomplete datasets in the accessibility scale

| Variable | Accessibility scale complete (n = 307) | Accessibility scale incomplete (n = 46) | p-value |
| --- | --- | --- | --- |
| Gender, *n* (%)  Female  Male | 173 (87.8%)  134 (85.9%) | 24 (12.2%)  22 (14.1%) | .595^a^ |
| Mean age in years*, *M (SD)* | 53.5 (9.1) | 53.5 (9.0) | .996^b^ |
| Migration, *n (%)*  No (German)  Yes  Insufficient information | 280 (88.6%)  22 (73.3%)  5 (71.4%) | 36 (11.4%)  8 (26.7%)  2 (28.6%) | .038^c^ |
| Education*, *n (%)*  Basic Education (up to 9 years)  Medium Education (10-11 years)  Higher Education (12 years and over) | 99 (86.8%)  122 (88.4%)  86 (86.0%) | 15 (13.2)  16 (11.6%)  14 (14.0%) | .851^a^ |
| Reason for Consultation, *n (%)*  Health Check  Disease Management for Diabetes | 294 (87.2%)  13 (81.3%) | 43 (12.8%)  3 (18.8%) | .449^c^ |
| Number of risk factors besides age*, *M (SD)* | 0.6 (0.8) | 0.6 (0.8) | .930^b^ |
| Contact with arriba prior to examination, *n (%)*  Yes  No | 50 (92.6%)  257 (86.0%) | 4 (7.4%)  42 (14.0%) | .182^a^ |

^a^= *χ^2^*-test; ^b^ = t-test; ^c^ = Fisher’s exact test
