# Supplementary material for: The effect of a new lifetime-cardiovascular-risk display on patients’ motivation to participate in shared decision-making
Source: BMC Fam Pract. 2018 Jun 9;19:84. doi: 10.1186/s12875-018-0766-x (PMC5994251; doi:10.1186/s12875-018-0766-x)
Supplement: Supplementary file 2 — Table S3. Comparison of patients randomly receiving either the low, medium or high cardiovascular risk case vignette. (DOCX 13 kb) [file 12875_2018_766_MOESM2_ESM.docx]

Supplemental table 3 Comparison of patients randomly receiving either the low, medium or high cardiovascular risk case vignette

| Variable | Low risk  (n = 122) | Medium risk  (n = 113) | High risk  (n = 118) | p-value |
| --- | --- | --- | --- | --- |
| Gender, *n* (%)  Female  Male | 63 (32.0%)  59 (37.8%) | 65 (33.0%)  48 (30.8%) | 69 (35.0%)  49 (31.4%) | .513 |
| Mean age in years*, *M (SD)* | 53.4 (9.5) | 54.2 (8.6) | 53.0 (9.0) | .568^b^ |
| Migration, *n (%)*  No (German)  Yes  Insufficient information | 106 (33.5%)  14 (46.7%)  2 (28.6%) | 105 (33.2%)  6 (20.0%)  2 (28.6%) | 105 (33.2%)  10 (33.3%)  3 (42.9%) | .240^a^ |
| Education*, *n (%)*  Basic Education (up to 9 years)  Medium Education (10-11 years)  Higher Education (12 years and over) | 41 (36.0%)  51 (37.0%)  30 (30.0%) | 35 (30.7%)  41 (29.7%)  37 (37.0%) | 38 (33.3%)  46 (33.3%)  33 (33.0%) | .744^a^ |
| Reason for Consultation, *n (%)*  Health Check  Disease Management for Diabetes | 118 (35.0%)  4 (25.0%) | 105 (31.2%)  8 (50.0%) | 114 (33.8%)  4 (25.0%) | .287^a^ |
| Number of risk factors besides age*, *M (SD)* | 0.5 (0.8) | 0.6 (0.8) | 0.6 (0.9) | .804^b^ |
| Contact with arriba prior to examination, *n (%)*  Yes  No | 20 (37.0%)  102 (34.1%) | 17 (31.5%)  96 (32.1%) | 17 (31.5%)  101 (33.8%) | .909^a^ |

^a^= *χ^2^*-test; ^b^ = t-test; ^c^ = Fisher’s exact test
